# Use of the EpiDerm^TM^ 3D reconstructed skin micronucleus assay for fragrance materials

**DOI:** 10.1093/mutage/geab040

**Published:** 2021-11-30

**Authors:** Yax Thakkar, Holger Moustakas, Marilyn Aardema, Shambhu Roy, Stefan Pfuhler, Anne Marie Api

**Affiliations:** 1 Research Institute for Fragrance Materials, Woodcliff Lake, NJ, USA; 2 Marilyn Aardema Consulting LLC, Fairfield, OH, USA; 3 Millipore Sigma, Rockville, MD, USA; 4 The Procter & Gamble Company, Mason Business Centre, Mason, OH, USA

**Keywords:** 3DRSMN, fragrance materials, animal alternative, genotoxicity

## Abstract

In order to evaluate the utility of the 3D reconstructed skin micronucleus assay (3DRSMN) to assess clastogenic/aneugenic potential of the fragrance chemicals, a set of 22 fragrance materials were evaluated in 3DRSMN assay. These materials evaluated were also evaluated in an *in vitro* as well as *in vivo* micronucleus assay, conducted as per Organisation for Economic Co-operation and Development guidelines. The results of the RSMN assay were in 100% agreement with the *in vivo* micronucleus assay results. From this dataset, 18 materials were positive in an *in vitro* micronucleus assay but were negative in an *in vivo* micronucleus assay. All these 18 materials were also concluded to be negative in 3DRSMN assay, stressing the importance of the assay to help minimize misleading positive outcomes from the *in vitro* assay. Since the highest exposure for fragrances is through the dermal route, the RSMN assay fits the applicability domain for testing. Thus, RSMN assay is an important alternative to animal testing for characterization of the genotoxicity potential of fragrance materials.

## Introduction

As of March 2009, the seventh amendment to the EU Cosmetics Directive banned the use of *in vivo* genotoxicity studies for cosmetic ingredients [1]. Without *in vivo* tests, there is no way to determine if a positive *in vitro* genotoxicity test result represents a real hazard or is a “misleading” positive, as is known to occur with a high frequency in the traditional battery of *in vitro* genotoxicity studies [2, 3]. Cosmetic and fragrance materials with positive results in the standard *in vitro* genotoxicity assays would thus be prevented from further development. According to Ates *et al*. (2014), non-confirmed or “misleading” positive results occurred in up to 93% of *in vitro* genotoxicity test batteries with cosmetic ingredients [4]. To address this issue, genotoxicity tests in 3D reconstructed human skin models were developed as a non-animal follow-up assay to assess genotoxicity via a dermal route of exposure. 3D reconstructed skin (RS) models consist of a multilayered, differentiated model of the human epidermis that offers more physiologically relevant results for dermally exposed substances when compared with the standard *in vitro* micronucleus test, as is most common in cosmetic and fragrance products. The metabolizing capacity of RS models is representative of human skin [5–9], therefore is more biologically relevant for assessing hazard from dermal exposures compared to the induced exogenous rat liver S9 used in standard *in vitro* genotoxicity tests. Importantly, RS models have relatively little or absent Phase I activities, ­similar to human skin, and have significantly higher and measurable Phase II detoxification activities, both of which contribute to the increased relevance of RS models for genotoxicity assessment with dermal exposure [10, 11].

The 3D reconstructed skin micronucleus assay (RSMN) was developed as a follow-up test for materials that are positive in standard *in vitro* chromosome aberrations or micronucleus assays. Inter- and intra-laboratory validation studies for the RSMN assay, including a global validation ­effort by Cosmetics Europe, have shown both good reproducibility and predictivity of *in vivo* genotoxicity results, making this assay an excellent animal alternative follow-up to traditional *in vitro* studies [12–14]. The utility of the RSMN assay and development of an Organisation for Economic Co-operation and Development (OECD) guideline was endorsed by the International Working Group on Genetic Toxicology (IWGT) in meetings in 2009 [15] and 2017 [16].

Since the RSMN assay is promising, the Research Institute for Fragrance Materials (RIFM) investigated the performance of 22 fragrance materials in the assay. The Crème RIFM aggregate exposure model demonstrates that dermal exposure is the primary route of exposure for most fragrance products, which further emphasizes the strengths the RSMN assay can offer for this category of materials [17–19]. The intent of this study is to determine whether the experimental dataset for fragrance materials presented here supports the observed high predictivity of the RSMN assay when compared with *in vivo* genotoxicity also. Another aspect of interest that can be investigated is whether the data support the suggestion to eliminate the 48-hour exposure time to streamline the assay [16].

## Materials and methods

### Selection of materials

In order to evaluate the utility of the 3D skin micronucleus assay to assess clastogenic/aneugenic potential of the fragrance chemicals, a set of 22 fragrance materials (supplied at market quality by RIFM member companies) were selected (Table 1) based on two criteria.

**Table 1.** Summary table describing all genotoxicity data for materials

Material	CAS #	*In vitro* MNT	3D Skin MNT	*In vivo* MNT
sec-Butyl ethyl ether	2679-87-0	+	-	-
Cadinene	29350-73-0	+^a^	-	
2,3-Dihydro-1,1-dimethyl-1H-indene-ar-propanal	300371-33-9	Equivocal	-	
1,5-Dimethylbicyclo[3.2.1]octan-8-one-oxime	75147-23-8	+	-	-
2,2ʹ-(Dithiodimethylene)difuran	4437-20-1	+	-	-
Ethyl formate	109-94-4	+	-	-
2-Ethyl-1,3,3-trimethyl-2-norbornanol	18368-91-7	-	-	-
Furfuryl thioacetate	13678-68-7	+	-	-
Isobornyl methyl ether	5331-32-8	Equivocal	-	-for read-across^b^
Lauric Aldehyde	112-54-9	+	-	-
p-Methoxy cinnamaldehyde	1963-36-6	+	-	-
6-Methoxy-2,6-dimethylheptan-1-al	62439-41-2	+	-	-
2-Methyl-2-pentenal	623-36-9	+	-	-
Methyl beta-phenylglycidate	37161-74-3	+∗	-	-
Nona-2 trans- 6-cis-dienal	557-48-2	+	-	-
2-Octenoic acid, 4-ethyl-, (2Z)	60308-75-0	+	-	-
2-Octen-4-one	4643-27-0	+	-	-
4-Phenyl-3-buten-2-ol	17488-65-2	+	-	-
5-Phenylhex-3-en-2-one	60405-50-7	Equivocal	-	-for read-across^c^
4-Thujanol	546-79-2	+	-	-
3,3,5-Trimethylcyclohexaneacetic acid	3213-73-8	+	-	-
Veratraldehyde	120-14-9	+	-	-

Results did not meet all criteria for a positive.

Read-across analogue is 1-ethyl-3-methoxytricyclo[2.2.1.02,6]heptane (CAS # 31996-78-8).

Read-across analogue is 4-Phenyl-3-buten-2-one (CAS#122-57-6).

(1) Each material was tested in a GLP compliant *in vitro* micronucleus assay in accordance with OECD TG 487 [20–22] using human peripheral blood lymphocytes in both the presence and absence of an S9 fraction from the livers of male Sprague Dawley rats induced with Aroclor 1254 or phenobarbital intraperitoneally and β-naphthoflavone.
(2) Each material was tested in a GLP compliant *in vivo* micronucleus assay in accordance with OECD TG 474 [23–25] using groups of male and female Hsd:IRC (CD-1) mice or Han Wistar rats with the test material administered either 2, 3, or 4 times in corn oil or deionized water via oral gavage.

Since the focus of this study was to investigate the ability of the RMSN assay to address “misleading” positive *in vitro* micronucleus assay results, all of the materials except one had a positive or equivocal result in the *in vitro* micronucleus assay, and those that were tested in the *in vivo* micronucleus assay were all negative. Three materials, namely, isobornyl methyl ether, 2,3-Dihydro-1,1-dimethyl-1H-indene-ar-propana, and cadinene, which produced either an equivocal or biologically non-relevant positive outcome in the *in vitro* micronucleus assay, were not tested in an *in vivo* micronucleus study. Equivocal was defined as results meeting some but not all the criteria for a positive outcome (such as a statistically significant increase as determined by the Fisher’s Exact test, but negative for dose–response as determined by the Cochran-Armitage test). Biologically non-relevant positive results were defined, for example, if the assay resulted in inconsistent statistically significant increases across repeat assays and steep increases in cytotoxicity at the concentrations with statistically significant increases. Results for these tests are included in the summary Table 1.

### RSMN assay

The RSMN assay was conducted according to the protocol described by Dahl *et al*. [26]. Assays were conducted in compliance with OECD GLP guidelines, most of the studies were conducted at contract research organizations. In the dose range-finding assay, and the first main study, tissues were treated with a 2-day dosing regimen (48 hours harvest). In the confirmatory micronucleus assay, tissues were treated with a 3-day dosing regimen (72 hours harvest). Each chemical was tested in triplicate, using tissues generated from the same batch/skin donor. Cytotoxicity was measured by both relative binucleation and RVCC (relative viable cell count) and whichever was the more sensitive parameter (resulted in 50–60% cytotoxicity first) was used to select the maximum dose for the micronucleus assay. In all the studies, acetone was used as a vehicle control, and mitomycin C (MMC) was used as a positive control and produced a statistically significant response demonstrating the validity of the study.

## Results

The results for each material are described below.

### sec-Butyl ethyl ether (CAS# 2679-87-0)

**Figure 1:**
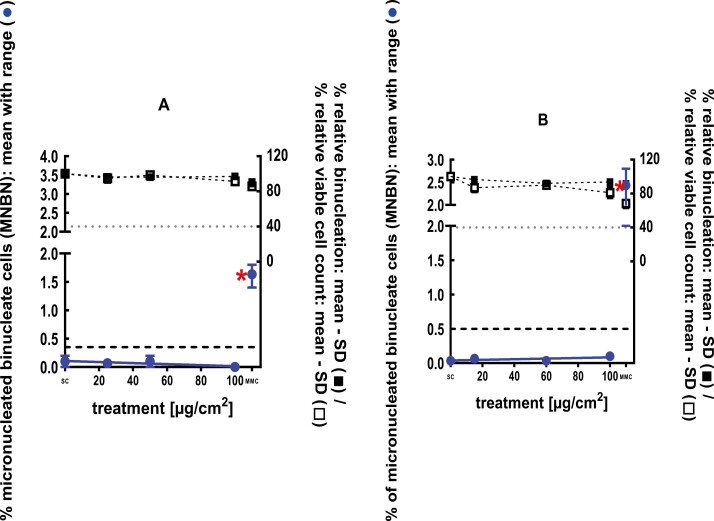
3Dskin RSMN results for sec-Butyl ethyl ether (CAS # 2679-87-0): (A) 48-hour treatment and (B) 72-hour treatment condition. The long-dashed black line shows the lab-specific 95%-quantile of the mean of the historical control range for the MNBN data, and the cutoff for excessive cytotoxicity (40%) is shown by the black dotted line. A red asterisk indicates a statistically significant increase in % MNBN vs. the SC (Fisher’s exact test; *P* < .05). Error bars shown depict the standard error of the mean.

The clastogenic activity of sec-butyl ethyl ether was evaluated in an *in vitro* micronucleus test conducted using human peripheral blood lymphocytes, at concentrations up to 1020 µg/ml in ethanol, in the presence and absence of metabolic activation. Statistically significant and dose-dependent increases in micronuclei induction were observed at doses 300 and 1020 µg/ml in the 4-hour treatment in the absence of S9. sec-Butyl ethyl ether was concluded to be positive for the induction of micronuclei in the *in vitro* mammalian cell micronucleus test [27]. A follow-up 3D skin and *in vivo* study were conducted to further evaluate the biological relevance of the positive *in vitro* results. In a GLP compliant RSMN assay, EpiDerm^TM^ tissues were treated with sec-butyl ethyl ether in acetone for 48 and 72 hours, at concentrations up to 100 mg/ml. sec-Butyl ethyl ether did not induce binucleated cells with micronuclei when tested up to cytotoxic levels, and therefore was concluded to be negative in the RSMN assay [28] (Fig. 1). A follow-up *in vivo* micronucleus study was also conducted in mice. The test material was administered in corn oil on two consecutive days at doses of 500, 1000, or 2000 mg/kg body weight via oral gavage to groups of male and female Hsd:ICR (CD-1) mice and euthanized at 48 hours. The test material did not induce a statistically significant increase in the incidence of micronucleated reticulocytes in the peripheral blood and was concluded to be negative in the *in vivo* micronucleus test [29] (Young, 2018; #75302).

### Cadinene (CAS# 29350-73-0)

**Figure 2:**
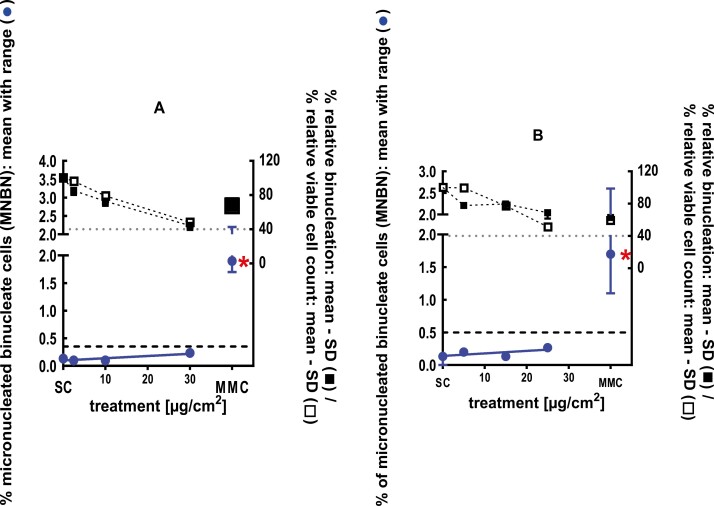
3Dskin RSMN results for Cadinene (CAS#29350-73-0): (A) 48-hour treatment and (B) 72-hour treatment condition. The long-dashed black line shows the lab-specific 95%-quantile of the mean of the historical control range for the MNBN data, and the cutoff for excessive cytotoxicity (40%) is shown by the black dotted line. A red asterisk indicates a statistically significant increase in % MNBN vs. the SC (Fisher’s exact test; *P* < .05). Error bars shown depict the standard error of the mean.

The clastogenic activity of cadinene was evaluated in an *in vitro* micronucleus test performed with human peripheral blood lymphocytes, at concentrations up to 1000 µg/ml in dimethyl formamide, in the presence and absence of metabolic activation. Cadinene did not induce binucleated cells with micronuclei when tested up to the cytotoxic level concentration in either the presence or absence of an S9 activation system in the 3-hour treatments. Cadinene did induce binucleated cells with micronuclei in the 24-hour treatment at doses of 41.3 and 58.1 µg/ml and was considered to be clastogenic with questionable biological relevance in the *in vitro* micronucleus test, as the increases observed were statistically significant but they were not dose-dependent or reproducible in every repeat assay [30]. A follow-up 3D skin study was evaluated to verify the *in vitro* results and their biological relevance. In a GLP compliant RSMN assay, EpiDerm^TM^ tissues were treated with cadinene in acetone for 48 and 72 hours, at concentrations up to 100 mg/ml. Cadinene did not induce binucleated cells with micronuclei when tested up to the maximum cytotoxic levels, and therefore was concluded to be negative in the RSMN assay [31] (Fig. 2).

### 2,3-Dihydro-1,1-dimethyl-1H-indene-ar-propanal (CAS# 300371-33-9)

**Figure 3:**
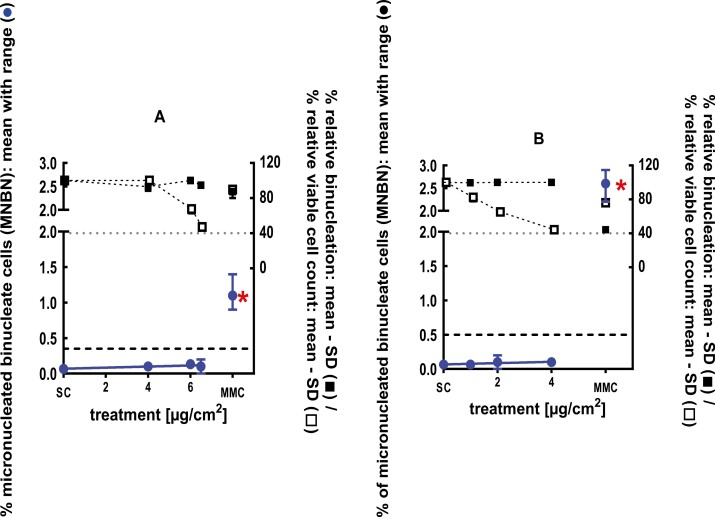
3Dskin RSMN results for 2,3-Dihydro-1,1-dimethyl-1H-indene-ar-propanal (CAS#300371-33-9): (A) 48-hour treatment and (B) 72-hour treatment condition. The long-dashed black line shows the lab-specific 95%-quantile of the mean of the historical control range for the MNBN data, and the thresholds for excessive cytotoxicity (40%) are shown by the black dotted line. A red asterisk indicates a statistically significant increase in % MNBN vs. the SC (Fisher’s exact test; *P* < .05). Error bars shown depict the standard error of the mean.

The clastogenic activity of 2,3-dihydro-1,1-dimethyl-1H-indene-ar-propanal was evaluated in an *in vitro* micronucleus performed with human peripheral blood lymphocytes, at concentrations up to 1300 µg/ml in dimethyl sulfoxide (DMSO), in the presence of and absence of metabolic ­activation. 2,3-Dihydro-1,1-dimethyl-1H-indene-ar-propanal did not induce binucleated cells with micronuclei when tested up to cytotoxic levels in non-activated 24-hour test systems. However, a statistically significant increase in micronuclei was observed at the 4-hour treatment period in the presence and absence of S9 metabolic activation at doses of 15 µg/ml in the presence of S9 and at doses of 7.5 and 35 µg/ml in the absence of S9. Despite these increases, a dose–response was not observed, and the study was concluded to be equivocal [32]. A follow-up 3D skin study was evaluated to verify the *in vitro* results and their biological relevance. In a GLP compliant RSMN assay, EpiDerm^TM^ tissues were treated with 2,3-dihydro-1,1-dimethyl-1H-indene-ar-propanal in acetone for 48 and 72 hours, at concentrations up to 12 mg/ml. 2,3-dihydro-1,1-dimethyl-1H-indene-ar-propanal did not induce binucleated cells with micronuclei when tested up to cytotoxic levels, and therefore was concluded to be negative in the RSMN assay [33] (Fig. 3).

### 1,5-Dimethylbicyclo[3.2.1]octan-8-one-oxime (CAS# 75147-23-8)

**Figure 4:**
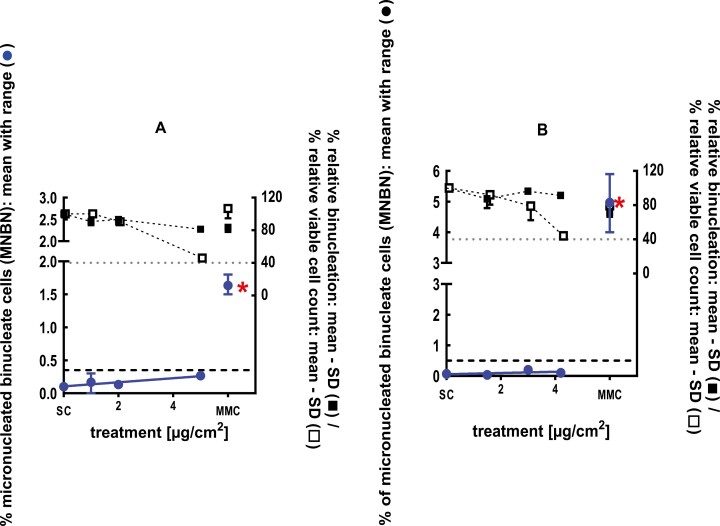
3Dskin RSMN results for 1,5-Dimethylbicyclo[3.2.1]octan-8-one-oxime (CAS# 75147-23-8): (A) 48-hour treatment and (B) 72-hour treatment condition. The long-dashed black line shows the lab-specific 95%-quantile of the mean of the historical control range for the MNBN data, and the thresholds for excessive cytotoxicity (40%) are shown by the black dotted line. A red asterisk indicates a statistically significant increase in % MNBN vs. the SC (Fisher’s exact test; *P* < .05). Error bars shown depict the standard error of the mean.

The clastogenic activity of 1,5-dimethylbicyclo[3.2.1]octan-8-one-oxime was evaluated in an *in vitro* micronucleus test performed with human peripheral blood lymphocytes, at concentrations up to 1482 µg/ml in DMSO, in the presence and absence of metabolic activation. Statistically significant and dose-dependent increases in micronuclei induction were observed at doses of 402 µg/ml in the 3-hour treatment in the presence of S9, 300 and 425 µg/ml in the 3-hour treatment in the absence of S9, and 47.3 µg/ml in the 24-hour treatment in the absence of S9. 1,5-Dimethylbicyclo[3.2.1]octan-8-one-oxime was concluded to be positive for the induction of micronuclei in the *in vitro* mammalian cell micronucleus test [34]. A follow-up 3D skin and *in vivo* study were evaluated to verify the *in vitro* results and their biological relevance. In a GLP-compliant RSMN assay, EpiDerm^TM^ tissues were treated with 1,5-dimethylbicyclo[3.2.1]octan-8-one-oxime for 48 and 72 hours, at concentrations up to 90 mg/ml. 1,5-Dimethylbicyclo[3.2.1]octan-8-one-oxime ether did not induce binucleated cells with micronuclei when tested up to cytotoxic levels, and therefore was concluded to be negative in the RSMN assay [35] (Fig. 4). A follow-up *in vivo* micronucleus study was also conducted in mice. The test material was administered in corn oil on two consecutive days at doses of 125, 250, or 500 mg/kg body weight via oral gavage to groups of male and female Hsd:ICR (CD-1) mice and euthanized at 48 hours. The test material did not induce a statistically significant increase in the incidence of micronucleated reticulocytes in the peripheral blood and was concluded to be negative in the *in vivo* micronucleus test [36].

### 2,2ʹ-(Dithiodimethylene)difuran (CAS# 4437-20-1)

**Figure 5:**
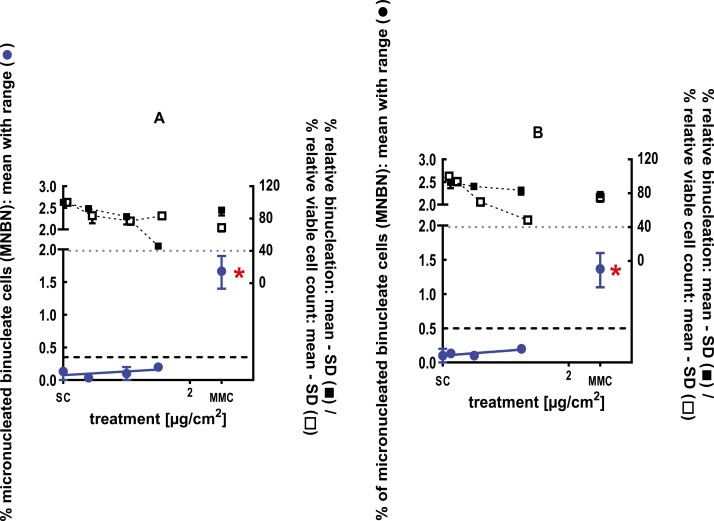
3Dskin RSMN results for 2,2ʹ-(Dithiodimethylene)difuran (CAS#4437-20-1): (A) 48-hour treatment and (B) 72-hour treatment condition. The long-dashed black line shows the lab-specific 95%-quantile of the mean of the historical control range for the MNBN data, and the thresholds for excessive cytotoxicity (40%) are shown by the black dotted line. A red asterisk indicates a statistically significant increase in % MNBN vs. the SC (Fisher’s exact test; *P* < .05). Error bars shown depict the standard error of the mean.

The clastogenic activity of 2,2ʹ-(dithiodimethylene)difuran was performed using human peripheral blood lymphocytes at concentrations up to 1000 µg/ml in DMSO. Statistically significant and dose-dependent increases in micronuclei induction were observed at doses of 29.5 and 32.8 µg/ml in the 3-hour treatment in the presence of S9 and at doses of 14.3, 19.5, and 29.4 µg/ml on the 3-hour treatment in the absence of S9 [37]. 2,2ʹ-(dithiodimethylene)difuran was considered to be positive for the induction of micronuclei in the *in vitro* mammalian cell micronucleus test. A follow-up 3D skin and *in vivo* study were evaluated to verify the *in vitro* results and their biological relevance. In a GLP-compliant RSMN assay, EpiDerm^TM^ tissues were treated with 2,2ʹ-(dithiodimethylene)difuran in DMSO for 48 and 72 hours, at concentrations up to 1.25 mg/ml. 2,2ʹ-(dithiodimethylene)difuran did not induce binucleated cells with micronuclei when tested up to cytotoxic levels, and therefore was concluded to be negative for the induction of micronuclei in the RSMN assay [38] (Fig. 5). A follow-up *in vivo* micronucleus study was also conducted in mice. The test material was administered in corn oil via oral gavage on two consecutive days at doses of 62.5, 125, or 250 mg/kg body weight to groups of male and female HSD:ICR (CD-1) mice and euthanized at 48 hours. The test material did not induce a significant increase in the incidence of micronucleated reticulocytes in the peripheral blood and was considered to be negative in the *in vivo* micronucleus test [39].

### Ethyl formate (CAS# 109-94-4)

**Figure 6:**
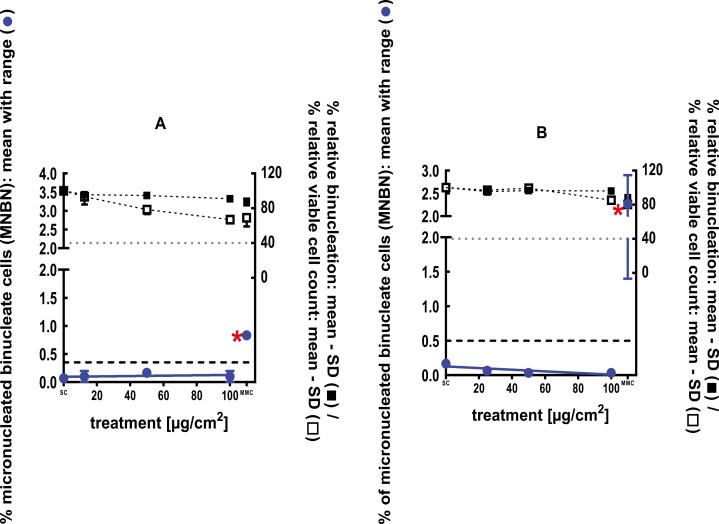
3Dskin RSMN results for Ethyl formate (CAS# 109-94-4): (A) 48-hour treatment and (B) 72-hour treatment condition. The long-dashed black line shows the lab-specific 95%-quantile of the mean of the historical control range for the MNBN data, and the thresholds for excessive cytotoxicity (40%) are shown by the black dotted line. A red asterisk indicates a statistically significant increase in % MNBN vs. the SC (Fisher’s exact test; *P* < .05). Error bars shown depict the standard error of the mean.

The clastogenic activity of ethyl formate was evaluated in an *in vitro* micronucleus test conducted using human peripheral blood lymphocytes, at concentrations up to 741 µg/ml in DMSO, in the presence and absence of metabolic activation. Statistically significant and dose-dependent increases in micronuclei induction were observed at doses of 596 and 741 µg/ml in the 3-hour treatment in the absence of S9, doses of 479, 596, and 741 µg/ml in the 3-hour treatment in the presence of S9, and doses of 554 µg/ml in the 24-hour treatment in the absence of S9. Ethyl formate was concluded to be positive for the induction of micronuclei in the *in vitro* mammalian cell micronucleus test [40]. A follow-up 3D skin and *in vivo* study were evaluated to verify the *in vitro* results and their biological relevance. In a GLP compliant RSMN assay, EpiDerm^TM^ tissues were treated with ethyl formate in ethanol for 48 and 72 hours, at concentrations up to 100 mg/ml. Ethyl formate did not induce binucleated cells with micronuclei when tested up to cytotoxic levels, and therefore was concluded to be negative in the RSMN assay [41] (Fig. 6). A follow-up *in vivo* micronucleus study was also conducted in mice. The test material was administered in corn oil on two consecutive days at doses of 500, 1000, or 2000 mg/kg body weight via oral gavage to groups of male and female Hsd:ICR (CD-1) mice and euthanized at 48 hours. The test material did not induce a statistically significant increase in the incidence of micronucleated reticulocytes in the peripheral blood and was concluded to be negative in the *in vivo* micronucleus test [42].

### 2-ethyl-1,3,3-trimethyl-2-norbornanol (CAS# 18368-91-7)

**Figure 7:**
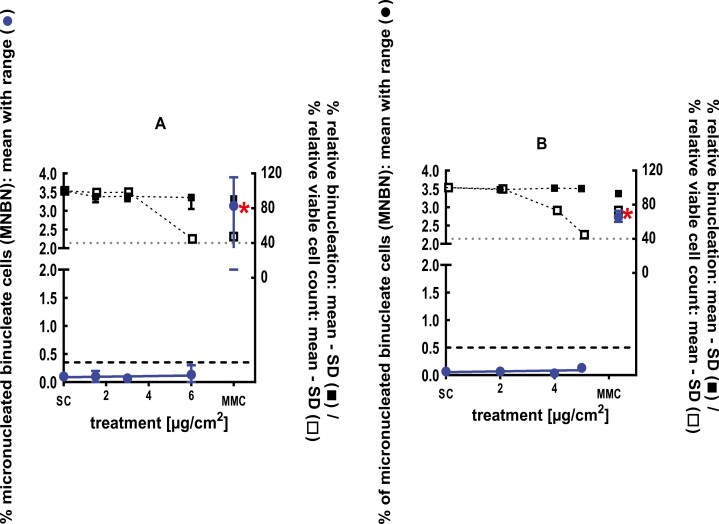
3Dskin RSMN results for 2-ethyl-1,3,3-trimethyl-2-norbornanol (CAS#18368-91-7): (A) 48-hour treatment and (B) 72-hour treatment condition. The long-dashed black line shows the lab-specific 95%-quantile of the mean of the historical control range for the MNBN data, and the thresholds for excessive cytotoxicity (40%) are shown by the black dotted line. A red asterisk indicates a statistically significant increase in % MNBN vs. the SC (Fisher’s exact test; *P* < .05). Error bars shown depict the standard error of the mean.

The clastogenic activity of 2-ethyl-1,3,3-trimethyl-2-norbornanol was evaluated in an *in vitro* micronucleus test conducted using human peripheral blood lymphocytes, at concentrations up to 1823 µg/ml in DMSO, in the presence and absence of metabolic activation. No statistically significant and dose-dependent increases in micronuclei induction were observed when tested up to cytotoxic levels concentration in either the presence or absence of an S9 activation system. 2-ethyl-1,3,3-trimethyl-2-norbornanol was concluded to be negative for the induction of micronuclei in the *in vitro* mammalian cell micronucleus test [43]. A follow-up 3D skin and *in vivo* study were evaluated to verify the *in vitro* results and their biological relevance. In a GLP compliant RSMN assay, EpiDerm^TM^ tissues were treated with 2-ethyl-1,3,3-trimethyl-2-norbornanol in acetone for 48 and 72 hours, at concentrations up to 6 mg/ml. 2-ethyl-1,3,3-trimethyl-2-norbornanol did not induce binucleated cells with micronuclei when tested up to cytotoxic levels, and therefore was concluded to be negative in the RSMN assay [44] (Fig. 7). A follow-up combined *in vivo* COMET/micronucleus study was also conducted in mice. The test material was administered in corn oil on four consecutive days at doses of 125, 250, or 500 mg/kg body weight via oral gavage to groups of male and female Hsd:ICR (CD-1) mice and euthanized at 3–4 hours post-last dose. The test material did not induce a statistically significant increase in the incidence of micronucleated reticulocytes in the blood or induce a significant increase in DNA damage in the liver and was concluded to be negative in the combined *in vivo* COMET/micronucleus test [45].

### Furfuryl thioacetate (CAS# 13678-68-7)

**Figure 8:**
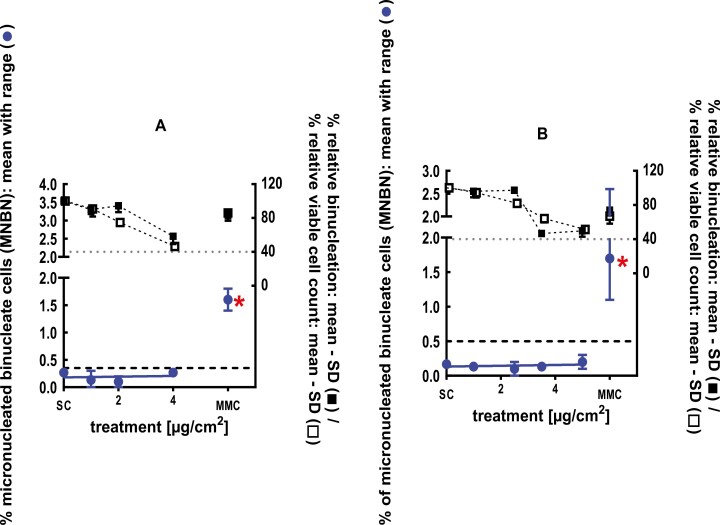
3Dskin RSMN results for Furfuryl thioacetate (CAS#13678-68-7): (A) 48-hour treatment and (B) 72-hour treatment condition. The long-dashed black line shows the lab-specific 95%-quantile of the mean of the historical control range for the MNBN data, and the thresholds for excessive cytotoxicity (40%) are shown by the black dotted line. A red asterisk indicates a statistically significant increase in % MNBN vs. the SC (Fisher’s exact test; *P* < .05). Error bars shown depict the standard error of the mean.

The clastogenic activity of furfuryl thioacetate was evaluated in an *in vitro* micronucleus test conducted using human peripheral blood lymphocytes, at concentrations up to 1000 µg/ml in DMSO, in the presence and absence of metabolic activation. Statistically significant and dose-dependent increases in micronuclei induction were observed at doses of 48.4 and 69.8 µg/ml in the 3-hour treatment in the absence of S9 and doses of 48.4, 59.7, and 69.8 µg/ml in the 3-hour treatment in the presence of S9. Furfuryl thioacetate was concluded to be positive for the induction of micronuclei in the *in vitro* mammalian cell micronucleus test [46]. A follow-up 3D skin and *in vivo* study were evaluated to verify the *in vitro* results and their biological relevance. In a GLP compliant RSMN assay, EpiDerm^TM^ tissues were treated with furfuryl thioacetate in acetone for 48 and 72 hours, at concentrations up to 5 mg/ml. Furfuryl thioacetate did not induce binucleated cells with micronuclei when tested up to cytotoxic levels, and therefore was concluded to be negative in the RSMN assay [47] (Fig. 8). A follow-up *in vivo* micronucleus study was also conducted in mice. The test material was administered in corn oil on two consecutive days at doses of 31.3, 62.5, and 125 mg/kg body weight via oral gavage to groups of male and female Hsd:ICR (CD-1) mice and euthanized at 48 hours. The test material did not induce a statistically significant increase in the incidence of micronucleated reticulocytes in the peripheral blood and was concluded to be negative in the *in vivo* micronucleus test [48].

### Isobornyl methyl ether (CAS# 5331-32-8)

**Figure 9:**
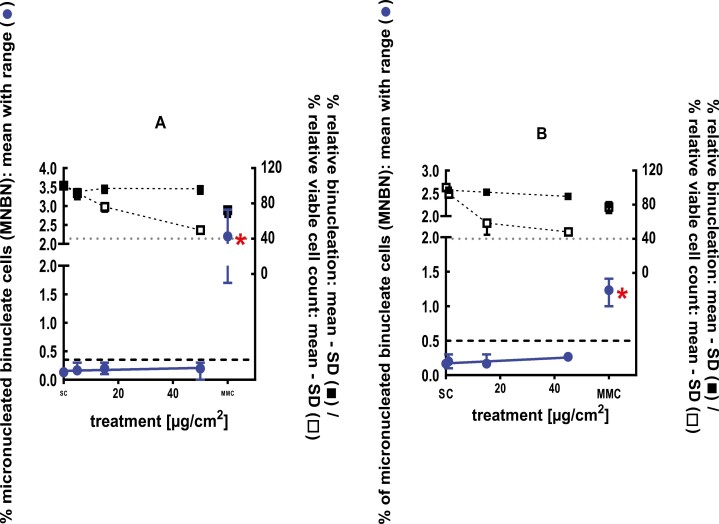
3Dskin RSMN results for Isobornyl methyl ether (CAS#5331-32-8): (A) 48-hour treatment and (B) 72-hour treatment condition. The long-dashed black line shows the lab-specific 95%-quantile of the mean of the historical control range for the MNBN data, and the thresholds for excessive cytotoxicity (40%) are shown by the black dotted line. A red asterisk indicates a statistically significant increase in % MNBN vs. the SC (Fisher’s exact test; *P* < .05). Error bars shown depict the standard error of the mean.

The clastogenic activity of isobornyl methyl ether was evaluated in an *in vitro* micronucleus test conducted using human peripheral blood lymphocytes, at concentrations up to 1680 µg/ml in ethanol, in the presence and absence of metabolic activation. Statistically significant and dose-dependent increases in micronuclei induction were observed at doses of 70 and 80 µg/ml in the 4-hour treatment in the presence of S9. Although the induced values (1.0 and 0.8%, respectively) were within the historical control range (0.0–1.5%), they fell outside of the 95% control range of historical control data (upper limit of 95% control range = 0.0–0.78%). In a repeat assay, statistically significant increases in micronuclei induction were observed at a dose of 80 µg/ml in the 4-hour treatment in the presence of S9. While the increase (1.1%) was outside the 95% control range of historical control data, the increase was not dose-dependent. Due to this, isobornyl methyl ether was concluded to be equivocal for the induction of micronuclei in the *in vitro* mammalian cell micronucleus test [49]. A follow-up 3D skin and *in vivo* study were evaluated to verify the *in vitro* results and their biological relevance. In a GLP compliant RSMN assay, EpiDerm^TM^ tissues were treated with isobornyl methyl ether in acetone for 48 and 72 hours, at concentrations up to 70 mg/ml. Isobornyl methyl ether did not induce binucleated cells with micronuclei when tested up to cytotoxic levels, and therefore was concluded to be negative in the RSMN assay [50] (Fig. 9). As an additional weight of evidence, a follow-up *in vivo* micronucleus study from read-across material 1-ethyl-3-methoxytricyclo[2.2.1.02,6]heptane (CAS # 31996-78-8) was also investigated. 1-ethyl-3-methoxytricyclo[2.2.1.02,6]heptane was administered in propylene glycol as a single dose of 3519 mg/kg body weight via intra gastric gavage to groups of male and female Hsd:ICR (CD-1) mice and euthanized at 24, 48, or 72 hours. 1-ethyl-3-methoxytricyclo[2.2.1.02,6]heptane did not induce a statistically significant increase in the incidence of micronucleated reticulocytes in the bone marrow and was concluded to be negative in the *in vivo* micronucleus test, and this result can be extended to isobornyl methyl ether [51].

### Lauric aldehyde (CAS # 112-54-9)

**Figure 10:**
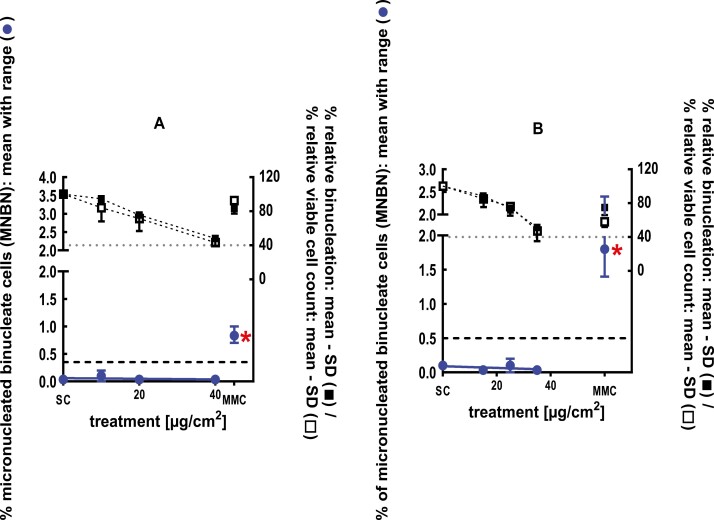
3Dskin RSMN results for Lauric Aldehyde (CAS # 112-54-9): (A) 48-hour treatment and (B) 72-hour treatment condition. The long-dashed black line shows the lab-specific 95%-quantile of the mean of the historical control range for the MNBN data, and the thresholds for excessive cytotoxicity (40%) are shown by the black dotted line. A red asterisk indicates a statistically significant increase in % MNBN vs. the SC (Fisher’s exact test; *P* < .05). Error bars shown depict the standard error of the mean.

The clastogenic activity of lauric aldehyde was evaluated in an *in vitro* micronucleus test conducted using human peripheral blood lymphocytes, at concentrations up to 1840 µg/ml in tetrahydrofuran, in the presence and absence of metabolic activation. Statistically significant and dose-dependent increases in micronuclei induction were observed at a dose of 30 µg/ml in the 4-hour treatment in the absence of S9 and doses of 20 and 35 µg/ml in the 4-hour treatment in the presence of S9. Lauric aldehyde was concluded to be positive for the induction of micronuclei in the *in vitro* mammalian cell micronucleus test [52]. A follow-up 3D skin and *in vivo* study were evaluated to verify the *in vitro* results and their biological relevance. In a GLP compliant RSMN assay, EpiDerm^TM^ tissues were treated with lauric aldehyde in acetone for 48 and 72 hours, at concentrations up to 100 mg/ml. Lauric aldehyde did not induce binucleated cells with micronuclei when tested up to cytotoxic levels, and therefore was concluded to be negative in the RSMN assay [53] (Fig. 10). A follow-up *in vivo* micronucleus study was also conducted in mice. The test material was administered in corn oil on two consecutive days at doses of 500, 1000, or 2000 mg/kg body weight via oral gavage to groups of male and female Hsd:ICR (CD-1) mice and euthanized at 48 hours. The test material did not induce a statistically significant increase in the incidence of micronucleated reticulocytes in the peripheral blood and was concluded to be negative in the *in vivo* micronucleus test [54].

### p-Methoxy cinnamaldehyde (CAS# 1963-36-6)

**Figure 11:**
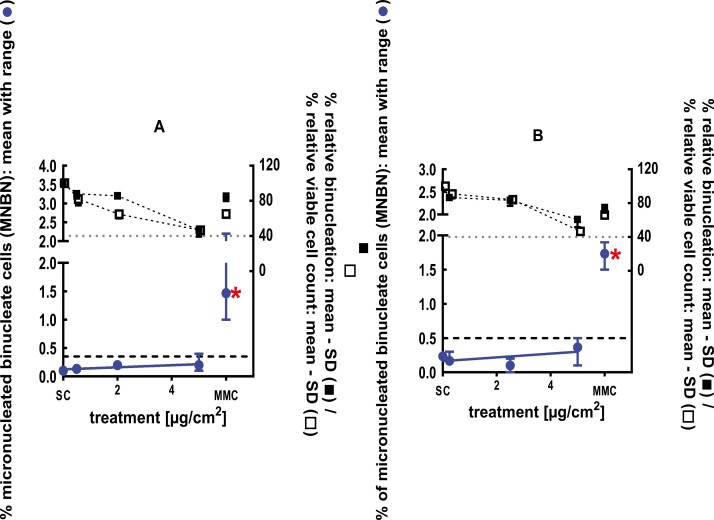
3Dskin RSMN results for p-Methoxy cinnamaldehyde (CAS#1963-36-6): (A) 48-hour treatment and (B) 72-hour treatment condition. The long-dashed black line shows the lab-specific 95%-quantile of the mean of the historical control range for the MNBN data, and the thresholds for excessive cytotoxicity (40%) are shown by the black dotted line. A red asterisk indicates a statistically significant increase in % MNBN vs. the SC (Fisher’s exact test; *P* < .05). Error bars shown depict the standard error of the mean.

The clastogenic activity of p-methoxy cinnamaldehyde was evaluated in an *in vitro* micronucleus test conducted using human peripheral blood lymphocytes, at concentrations up to 1624 µg/ml in DMSO, in the presence and absence of metabolic activation. Statistically significant and dose-dependent increases in micronuclei induction were observed at doses of 75., 100, and 110 µg/ml in the 3-hour treatment in the absence of S9, a dose of 160 µg/ml in the 3-hour treatment in the presence of S9, and a dose of 110 µg/ml in the 24-hour treatment in the absence of S9. p-Methoxy cinnamaldehyde was concluded to be positive for the induction of micronuclei in the *in vitro* mammalian cell micronucleus test [55]. A follow-up 3D skin and *in vivo* study were evaluated to verify the *in vitro* results and their biological relevance. In a GLP compliant RSMN assay, EpiDerm^TM^ tissues were treated with p-methoxy cinnamaldehyde in acetone for 48 and 72 hours, at concentrations up to 5 mg/ml. p-Methoxy cinnamaldehyde did not induce binucleated cells with micronuclei when tested up to cytotoxic levels, and therefore was concluded to be negative in the RSMN assay [56] (Fig. 11). A follow-up combined *in vivo* COMET/micronucleus study was also conducted in mice. The test material was administered in 1% Methylcellulose (400cPS) in deionized water on four consecutive days at doses of 500, 1000, or 2000 mg/kg body weight via oral gavage to groups of male and female Hsd:ICR (CD-1) mice and euthanized at 3–4 hours post-last dose. The test material did not induce a statistically significant increase in the incidence of micronucleated reticulocytes in the blood or induce a significant increase in DNA damage in the liver and was concluded to be negative in the combined *in vivo* COMET/micronucleus test [57].

### 6-Methoxy-2,6-dimethylheptan-1-al (CAS# 62439-41-2)

**Figure 12:**
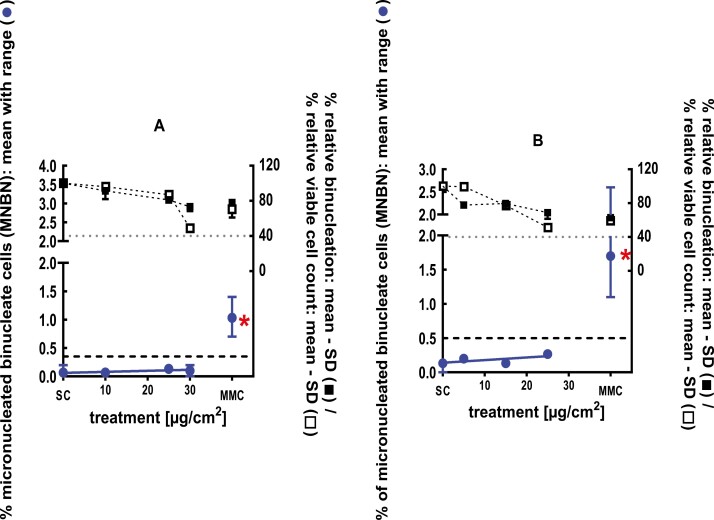
3Dskin RSMN results for 6-Methoxy-2,6-dimethylheptan-1-al (CAS # 62439-41-2): (A) 48-hour treatment and (B) 72-hour treatment condition. The long-dashed black line shows the lab-specific 95%-quantile of the mean of the historical control range for the MNBN data, and the thresholds for excessive cytotoxicity (40%) are shown by the black dotted line. A red asterisk indicates a statistically significant increase in % MNBN vs. the SC (Fisher’s exact test; *P* < .05). Error bars shown depict the standard error of the mean.

The clastogenic activity of 6-methoxy-2,6-dimethylheptan-1-al was evaluated in an *in vitro* micronucleus test ­conducted using human peripheral blood lymphocytes, at concentrations up to 1723 µg/ml in DMSO, in the presence and absence of metabolic activation. Statistically significant and dose-dependent increases in micronuclei induction were observed at doses of 1245, 1465, and 1723 µg/ml in the 3-hour treatment in the absence of S9 and a doses of 1723 µg/ml in the 3-hour treatment in the presence of S9. 6-Methoxy-2,6-dimethylheptan-1-al was concluded to be positive for the induction of micronuclei in the *in vitro* mammalian cell micronucleus test [58]. A follow-up 3D skin and *in vivo* study were evaluated to verify the *in vitro* results and their biological relevance. In a GLP compliant RSMN assay, EpiDerm^TM^ tissues were treated with 6-methoxy-2,6-dimethylheptan-1-al in acetone for 48 and 72 hours, at ­concentrations up to 45 mg/ml. 6-Methoxy-2,6-dimethylheptan-1-al did not induce binucleated cells with micronuclei when tested up to cytotoxic levels, and therefore was concluded to be negative in the RSMN assay [59] (Fig. 12). A follow-up *in vivo* micronucleus study was also conducted in mice. The test material was administered in corn oil on two consecutive days at doses of 500, 1000, or 2000 mg/kg body weight via oral gavage to groups of male and female Hsd:ICR (CD-1) mice and euthanized at 48 hours. The test material did not induce a statistically significant increase in the incidence of micronucleated reticulocytes in the peripheral blood and was concluded to be negative in the *in vivo* micronucleus test [60].

### 2-Methyl-2-pentenal (CAS# 623-36-9)

**Figure 13:**
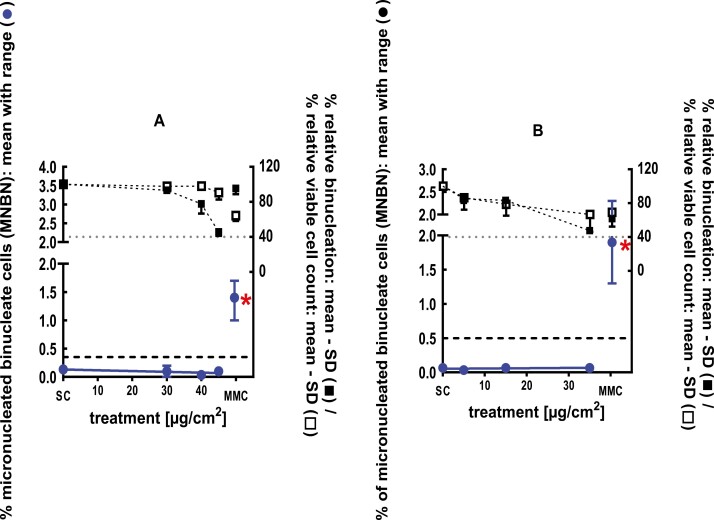
3Dskin RSMN results for 2-Methyl-2-pentenal (CAS#623-36-9): (A) 48-hour treatment and (B) 72-hour treatment condition. The long-dashed black line shows the lab-specific 95%-quantile of the mean of the historical control range for the MNBN data, and the thresholds for excessive cytotoxicity (40%) are shown by the black dotted line. A red asterisk indicates a statistically significant increase in % MNBN vs. the SC (Fisher’s exact test; *P* < .05). Error bars shown depict the standard error of the mean.

The clastogenic activity of 2-methyl-2-pentenal was evaluated in an *in vitro* micronucleus test conducted using human peripheral blood lymphocytes, at concentrations up to 981 µg/ml in DMSO, in the presence and absence of metabolic activation. Statistically significant and dose-dependent increases in micronuclei induction were observed at a dose of 500 µg/ml in the 4-hour treatment in the presence and absence of S9 and a dose of 125 µg/ml in the 24-hour treatment in the absence of S9. 2-Methyl-2-pentenal was concluded to be positive for the induction of micronuclei in the *in vitro* mammalian cell micronucleus test [61]. A follow-up 3D skin and *in vivo* study were evaluated to verify the *in vitro* results and their biological relevance. In a GLP compliant RSMN assay, EpiDerm^TM^ tissues were treated with 2-methyl-2-pentenal in acetone for 48 and 72 hours, at concentrations up to 45 mg/ml. 2-Methyl-2-pentenal did not induce binucleated cells with micronuclei when tested up to cytotoxic levels, and therefore was concluded to be negative in the RSMN assay [62] (Fig. 13). A follow-up combined *in vivo* COMET/micronucleus study was also conducted in mice. The test material was administered in corn oil on three consecutive days at doses of 350, 700, or 1400 mg/kg body weight via oral gavage to groups of male and female Han Wistar rats and euthanized at 3–4 hours post-last dose. The test material did not induce a statistically significant increase in the incidence of micronucleated reticulocytes in the bone marrow or induce a significant increase in DNA damage in the liver and was concluded to be negative in the combined *in vivo* COMET/micronucleus test [63].

### Methyl beta-phenylglycidate (CAS# 37161-74-3)

**Figure 14:**
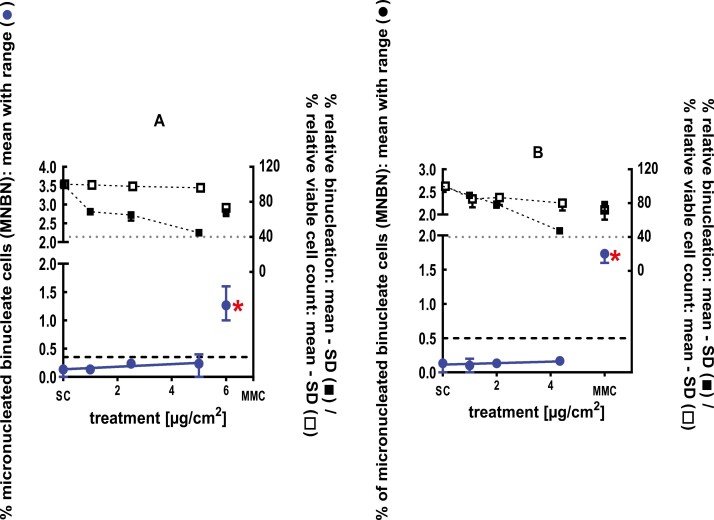
3Dskin RSMN results for Methyl beta-phenylglycidate (CAS#37161-74-3): (A) 48-hour treatment and (B) 72-hour treatment condition. The long-dashed black line shows the lab-specific 95%-quantile of the mean of the historical control range for the MNBN data, and the thresholds for excessive cytotoxicity (40%) are shown by the black dotted line. A red asterisk indicates a statistically significant increase in % MNBN vs. the SC (Fisher’s exact test; *P* < .05). Error bars shown depict the standard error of the mean.

The clastogenic activity of methyl beta-phenylglycidate was evaluated in an *in vitro* micronucleus test conducted using human peripheral blood lymphocytes, at concentrations up to 1782 µg/ml in DMSO, in the presence and absence of metabolic activation. Statistically significant increases in micronuclei induction were observed at doses of 581, 646, and 886 µg/ml in the 3-hour treatment in the presence of S9; however, no clear concentration related increase was observed. Methyl beta-phenylglycidate was concluded to be positive with questionable biological relevance for the induction of micronuclei in the *in vitro* mammalian cell micronucleus test [64]. A follow-up 3D skin and *in vivo* study were evaluated to verify the *in vitro* results and their biological relevance. In a GLP compliant RSMN assay, EpiDerm^TM^ tissues were treated with methyl beta-phenylglycidate in acetone for 48 and 72 hours, at concentrations up to 4.34 mg/ml. Methyl beta-phenylglycidate did not induce binucleated cells with micronuclei when tested up to cytotoxic levels, and therefore was concluded to be negative in the RSMN assay [65] (Fig. 14). A follow-up combined *in vivo* COMET/micronucleus study was also conducted in mice. The test material was administered in corn oil on four consecutive days at doses of 250, 500, or 1000 mg/kg body weight via oral gavage to groups of male and female Hsd:ICR (CD-1) mice and euthanized at 3–4 hours post-last dose. The test material did not induce a statistically significant increase in the incidence of micronucleated reticulocytes in the peripheral blood or induce a significant increase in DNA damage in the liver and was concluded to be negative in the combined *in vivo* COMET/micronucleus test [66].

### Nona-2 trans- 6-cis-dienal (CAS# 557-48-2)

**Figure 15:**
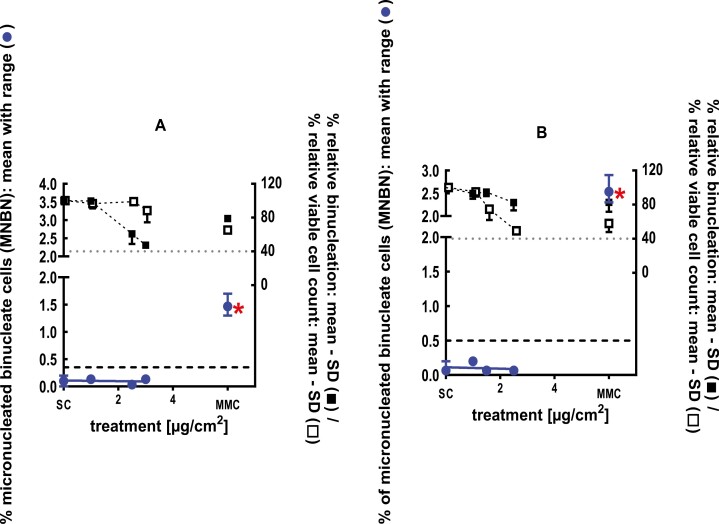
3Dskin RSMN results for Nona-2 trans- 6-cis-dienal (CAS# 557-48-2): (A) 48-hour treatment and (B) 72-hour treatment condition. The long-dashed black line shows the lab-specific 95%-quantile of the mean of the historical control range for the MNBN data, and the thresholds for excessive cytotoxicity (40%) are shown by the black dotted line. A red asterisk indicates a statistically significant increase in % MNBN vs. the SC (Fisher’s exact test; *P* < .05). Error bars shown depict the standard error of the mean.

The clastogenic activity of nona-2-*trans*-6-*cis*-dienal was evaluated in an *in vitro* micronucleus test conducted using human peripheral blood lymphocytes, at concentrations up to 60 µg/ml in DMSO, in the presence and absence of metabolic activation. Statistically significant and dose-dependent increases in micronuclei induction were observed at doses of 15 and 20 µg/ml in the 4-hour treatment in the absence of S9, at a dose of 40 µg/ml in the 4-hour treatment in the presence of S9, and at doses of 20 and 30 µg/ml in the 24-hour treatment in the absence of S9. Nona-2-*trans*-6-*cis*-dienal was concluded to be positive for the induction of micronuclei in the *in vitro* mammalian cell micronucleus test [67]. A follow-up 3D skin and *in vivo* study were evaluated to verify the *in vitro* results and their biological relevance. In a GLP compliant RSMN assay, EpiDerm^TM^ tissues were treated with nona-2-*trans*-6-*cis*-dienal in acetone for 48 and 72 hours, at concentrations up to 3 mg/ml. Nona-2-*trans*-6-*cis*-dienal did not induce binucleated cells with micronuclei when tested up to cytotoxic levels, and therefore was concluded to be negative in the RSMN assay [68] (Fig. 15). A follow-up combined *in vivo* COMET/micronucleus study was also conducted in rats. The test material was administered in corn oil on three consecutive days at doses of 175, 350, or 700 mg/kg body weight via oral gavage to groups of male and female Han Wistar rats and euthanized at 3–4 hours post-last dose. The test material did not induce a statistically significant increase in the incidence of micronucleated reticulocytes in the bone marrow or induce a significant increase in DNA damage in the liver and was concluded to be negative in the combined *in vivo* COMET/micronucleus test [69].

### 2-Octenoic acid, 4-ethyl-, (2E)- (CAS# 60308-76-1)

**Figure 16:**
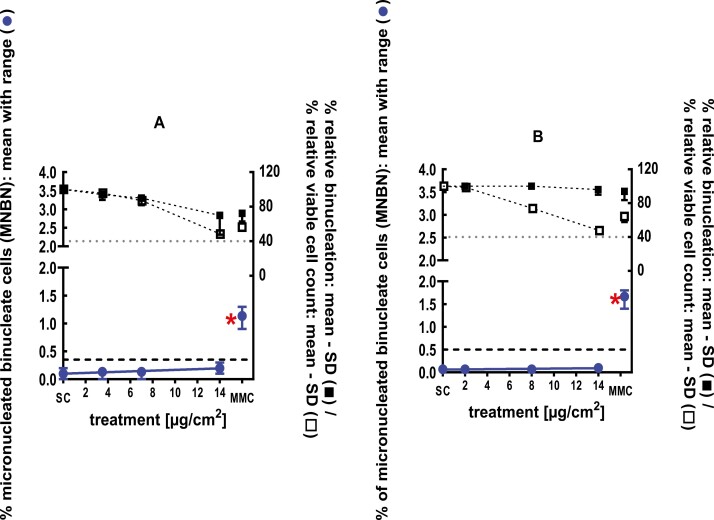
3Dskin RSMN results for 2-Octenoic acid, 4-ethyl-,(CAS# 60308-76-1): (A) 48-hour treatment and (B) 72-hour treatment condition. The long-dashed black line shows the lab-specific 95%-quantile of the mean of the historical control range for the MNBN data, and the thresholds for excessive cytotoxicity (40%) are shown by the black dotted line. A red asterisk indicates a statistically significant increase in % MNBN vs. the SC (Fisher’s exact test; *P* < .05). Error bars shown depict the standard error of the mean.

The clastogenic activity of 2-octenoic acid, 4-ethyl-, (2E)- was evaluated in an *in vitro* micronucleus test conducted using human peripheral blood lymphocytes, at concentrations up to 1704 µg/ml in DMSO, in the presence and absence of metabolic activation. Statistically significant and dose-dependent increases in micronuclei induction were observed at doses of 564, 658, and 692 µg/ml in the 3-hour treatment in the absence of S9 and at doses of 570 and 600 µg/ml in the 3-hour treatment in the presence of S9. 2-Octenoic acid, 4-ethyl-, (2E)- was concluded to be positive for the induction of micronuclei in the *in vitro* mammalian cell micronucleus test [70]. A follow-up 3D skin and *in vivo* study were evaluated to verify the *in vitro* results and their biological relevance. In a GLP compliant RSMN assay, EpiDerm^TM^ tissues were treated with 2-octenoic acid, 4-ethyl-, (2E)- in acetone for 48 and 72 hours, at concentrations up to 14 mg/ml. 2-Octenoic acid, 4-ethyl-, (2E)- did not induce binucleated cells with micronuclei when tested up to cytotoxic levels, and therefore was concluded to be negative in the RSMN assay [71] (Fig. 16). A follow-up combined *in vivo* COMET/micronucleus study was also conducted in mice. The test material was administered in corn oil on four consecutive days at doses of 250, 500, and 1000 mg/kg body weight via oral gavage to groups of male and female Hsd:ICR (CD-1) mice and euthanized at 3–4 hours post-last dose. The test material did not induce a statistically significant increase in the incidence of micronucleated reticulocytes in the blood for male or female mice or induce a significant increase in DNA damage in the liver for male mice. However, a statistically significant increase in DNA damage in the liver for female mice was observed at 1000 mg/ kg body weight. 2-Octenoic acid, 4-ethyl-, (2E)- was concluded to be negative in the combined *in vivo* COMET/micronucleus test in males and positive in the *in vivo* COMET assay for females [72].

### 2-Octen-4-one (CAS# 4643-27-0)

**Figure 17:**
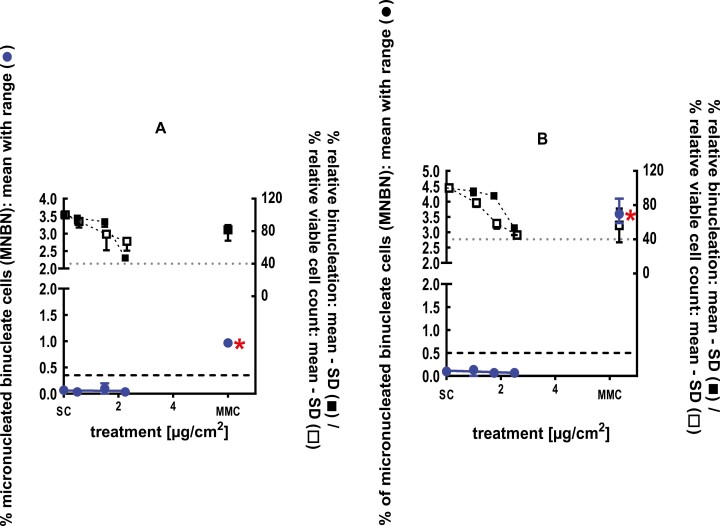
3Dskin RSMN results for 2-Octen-4-one (CAS#4643-27-0): (A) 48-hour treatment and (B) 72-hour treatment condition. The long-dashed black line shows the lab-specific 95%-quantile of the mean of the historical control range for the MNBN data, and the thresholds for excessive cytotoxicity (40%) are shown by the black dotted line. A red asterisk indicates a statistically significant increase in % MNBN vs. the SC (Fisher’s exact test; *P* < .05). Error bars shown depict the standard error of the mean.

The clastogenic activity of 2-octen-4-one was evaluated in an *in vitro* micronucleus test conducted using human peripheral blood lymphocytes, at concentrations up to 1265 µg/ml in DMSO, in the presence and absence of metabolic activation. Statistically significant and dose-dependent increases in micronuclei induction were observed at doses of 13.4, 16.5, and 20.4 µg/ml in the 3-hour treatment in the absence of S9 and at doses of 31.5 and 38.9 µg/ml in the 3-hour treatment in the presence of S9. 2-Octen-4-one was concluded to be positive for the induction of micronuclei in the *in vitro* mammalian cell micronucleus test [73]. A follow-up 3D skin and *in vivo* study were evaluated to verify the *in vitro* results and their biological relevance. In a GLP compliant RSMN assay, EpiDerm^TM^ tissues were treated with 2-octen-4-one in acetone for 48 and 72 hours, at concentrations up to 2.5 mg/ml. 2-Octen-4-one did not induce binucleated cells with micronuclei when tested up to cytotoxic levels, and therefore was concluded to be negative in the RSMN assay [74] (Fig. 17). A follow-up *in vivo* micronucleus study was also conducted in mice. The test material was administered in corn oil on two consecutive days at doses of 250, 500, or 1000 mg/kg body weight via oral gavage to groups of male and female Hsd:ICR (CD-1) mice and euthanized at 48 hours. The test material did not induce a statistically significant increase in the incidence of micronucleated reticulocytes in the peripheral blood and was concluded to be negative in the *in vivo* micronucleus test [75].

### 4-Phenyl-3-buten-2-ol (CAS# 17488-65-2)

**Figure 18:**
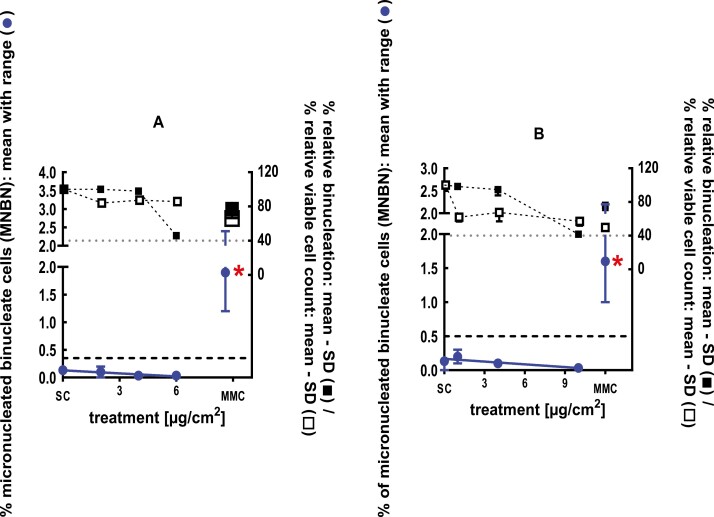
3Dskin RSMN results for 4-Phenyl-3-buten-2-ol (CAS#17488-65-2): (A) 48-hour treatment and (B) 72-hour treatment condition. The long-dashed black line shows the lab-specific 95%-quantile of the mean of the historical control range for the MNBN data, and the thresholds for excessive cytotoxicity (40%) are shown by the black dotted line. A red asterisk indicates a statistically significant increase in % MNBN vs. the SC (Fisher’s exact test; *P* < .05). Error bars shown depict the standard error of the mean.

The clastogenic activity of 4-phenyl-3-buten-2-ol was evaluated in an *in vitro* micronucleus test conducted using human peripheral blood lymphocytes, at concentrations up to 1000.00 mM (1482 µg/ml) in DMSO, in the presence and absence of metabolic activation. Statistically significant and dose-dependent increases in micronuclei induction were observed at doses of 666.67 mM (988 µg/ml) in the 3-hour treatment in the presence of S9. 4-Phenyl-3-buten-2-ol was concluded to be positive for the induction of micronuclei in the *in vitro* mammalian cell micronucleus test [76]. A follow-up 3D skin and *in vivo* study were evaluated to verify the *in vitro* results and their biological relevance. In a GLP compliant RSMN assay, EpiDerm^TM^ tissues were treated with 4-phenyl-3-buten-2-ol in acetone for 48 and 72 hours, at concentrations up to 14 mg/ml. 4-Phenyl-3-buten-2-ol did not induce binucleated cells with micronuclei when tested up to cytotoxic levels, and therefore was concluded to be negative in the RSMN assay [77] (Fig. 18). A follow-up *in vivo* micronucleus study was also conducted in mice. The test material was administered in corn oil on two consecutive days at doses of 500, 1000, or 2000 mg/kg body weight via oral gavage to groups of male and female Hsd:ICR (CD-1) mice and euthanized at 48 hours. The test material did not induce a statistically significant increase in the incidence of micronucleated reticulocytes in the peripheral blood and was concluded to be negative in the *in vivo* micronucleus test [78].

### 5-Phenylhex-3-en-2-one (CAS# 60405-50-7)

**Figure 19:**
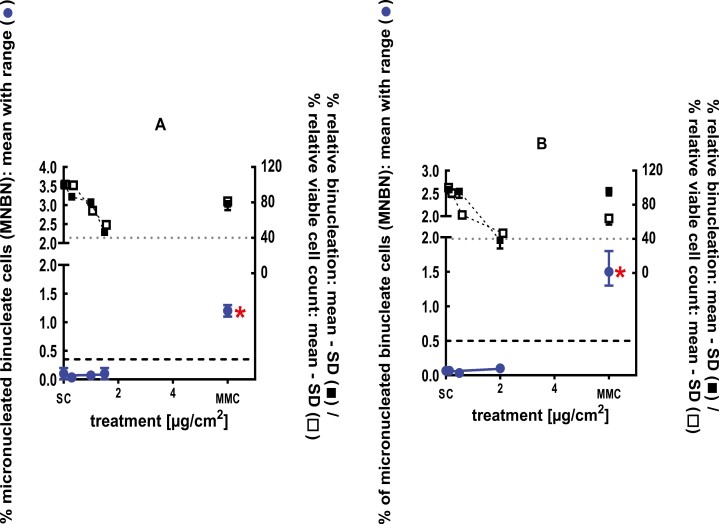
3Dskin RSMN results for 5-Phenylhex-3-en-2-one (CAS#60405-50-7): (A) 48-hour treatment and (B) 72-hour treatment condition. The long-dashed black line shows the lab-specific 95%-quantile of the mean of the historical control range for the MNBN data, and the thresholds for excessive cytotoxicity (40%) are shown by the black dotted line. A red asterisk indicates a statistically significant increase in % MNBN vs. the SC (Fisher’s exact test; *P* < .05). Error bars shown depict the standard error of the mean.

The clastogenic activity of 5-phenylhex-3-en-2-one was evaluated in an *in vitro* micronucleus test conducted using human peripheral blood lymphocytes, at concentrations up to 1740 µg/ml in DMSO, in the presence and absence of metabolic activation. Statistically significant and dose-dependent increases in micronuclei induction were observed at doses of 20, 22.5, and 25 µg/ml in the 24-hour treatment in the absence of S9 in an initial and confirmatory assay. However, while the initial assay was outside the 95% historical control range and the confirmatory assay was within the 95% historical control range, both assays were within the historical control range. Due to this, 5-Phenylhex-3-en-2-one was concluded to be equivocal for the induction of micronuclei in the *in vitro* mammalian cell micronucleus test [79]. A follow-up 3D skin and *in vivo* study were evaluated to verify the *in vitro* results and their biological relevance. In a GLP compliant RSMN assay, EpiDerm^TM^ tissues were treated with 5-phenylhex-3-en-2-one in acetone for 48 and 72 hours, at concentrations up to 2 mg/ml. 5-Phenylhex-3-en-2-one did not induce binucleated cells with micronuclei when tested up to cytotoxic levels, and therefore was concluded to be negative in the RSMN assay [80] (Fig. 19). As an additional weight of evidence, a combined *in vivo* COMET/micronucleus study from read-across material 4-phenyl-3-buten-2-one (CAS#122-57-6) was also available. The test material was administered in corn oil on three consecutive days at doses of 250, 500, or 1000 mg/kg body weight via oral gavage to groups of male and female Han Wistar rats and euthanized at 3–4 hours post-last dose. The test material did not induce a statistically significant increase in the incidence of micronucleated reticulocytes in the bone marrow or induce a significant increase in DNA damage in the liver and was concluded to be negative in the combined *in vivo* COMET/micronucleus test [81], and this result can be extended to 5-phenylhex-3-en-2-one due to the structural similarity with 4-phenyl-3-buten-2-one.

### 4-Thujanol (CAS# 546-79-2)

**Figure 20:**
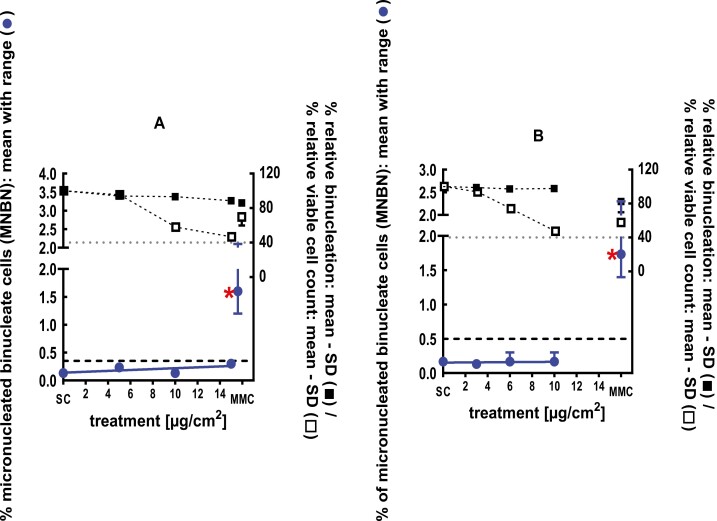
3Dskin RSMN results for 4-Thujanol (CAS# 546-79-2): A) 48-hour treatment and B) 72-hour treatment condition. The long-dashed black line shows the lab-specific 95%-quantile of the mean of the historical control range for the MNBN data, and the thresholds for excessive cytotoxicity (40%) are shown by the black dotted line. A red asterisk indicates a statistically significant increase in % MNBN vs. the SC (Fisher’s exact test; *P* < .05). Error bars shown depict the standard error of the mean.

The clastogenic activity of 4-thujanol was evaluated in an *in vitro* micronucleus test conducted using human peripheral blood lymphocytes, at concentrations up to 1540 µg/ml in DMSO, in the presence and absence of metabolic activation. Statistically significant and dose-dependent increases in micronuclei induction were observed at a dose of 630 µg/ml in the 4-hour treatment in the absence of S9. 4-Thujanol was concluded to be positive for the induction of micronuclei in the *in vitro* mammalian cell micronucleus test [82]. A follow-up 3D skin and *in vivo* study were evaluated to verify the *in vitro* results and their biological relevance. In a GLP compliant RSMN assay, EpiDerm^TM^ tissues were treated with 4-thujanol in acetone for 48 and 72 hours, at concentrations up to 2 mg/ml. 4-Thujanol did not induce binucleated cells with micronuclei when tested up to cytotoxic levels, and therefore was concluded to be negative in the RSMN assay [83] (Fig. 20). A follow-up combined *in vivo* COMET/micronucleus study was also conducted in mice. The test material was administered in corn oil on four consecutive days at doses of 250, 500, and 1000 mg/kg body weight via oral gavage to groups of male and female Hsd:ICR (CD-1) mice and euthanized at 3–4 hours post-last dose. The test material did induce a statistically significant increase in the incidence of micronucleated reticulocytes in the blood at 500 and 1000 mg/kg/day in males and did induce a significant increase in DNA damage in the liver at 500 and 1000 mg/kg/day in males and at 1000 mg/kg/day in females. However, these increases were within the historical control range, and therefore not considered to be biologically significant. 4-Thujanol was concluded to be negative in the combined *in vivo* COMET/micronucleus test [84].

### 3,3,5-Trimethylcyclohexaneacetic acid (CAS# 3213-73-8)

**Figure 21:**
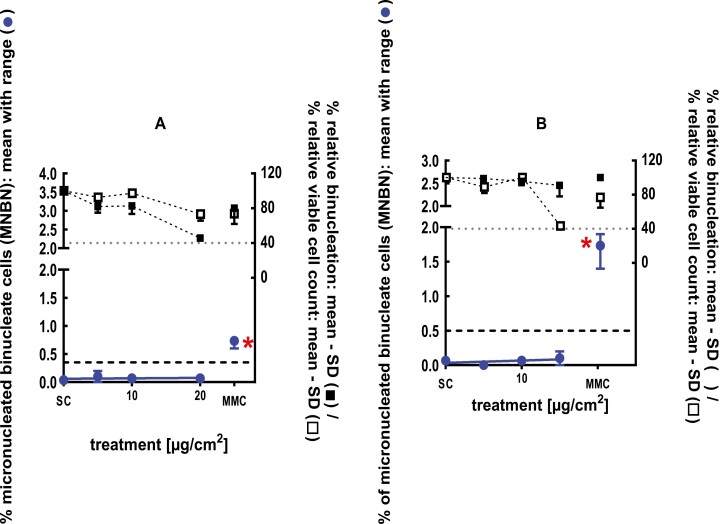
3Dskin RSMN results for 3,3,5-Trimethylcyclohexaneacetic acid (CAS#3213-73-8): (A) 48-hour treatment and (B) 72-hour treatment condition. The long-dashed black line shows the lab-specific 95%-quantile of the mean of the historical control range for the MNBN data, and the thresholds for excessive cytotoxicity (40%) are shown by the black dotted line. A red asterisk indicates a statistically significant increase in % MNBN vs. the SC (Fisher’s exact test; *P* < .05). Error bars shown depict the standard error of the mean.

The clastogenic activity of 3,3,5-trimethylcyclohexaneacetic acid was evaluated in an *in vitro* micronucleus test ­conducted using human peripheral blood lymphocytes, at concentrations up to 1840 µg/ml in DMSO, in the presence and absence of metabolic activation. Statistically significant and dose-dependent increases in micronuclei induction were observed at doses of 809 and 851 µg/ml in the 3-hour treatment in the presence of S9. 3,3,5-Trimethylcyclohexaneacetic acid was concluded to be positive for the induction of micronuclei in the *in vitro* mammalian cell micronucleus test [85]. A follow-up 3D skin and *in vivo* study were evaluated to verify the *in vitro* results and their biological relevance. In a GLP compliant RSMN assay, EpiDerm^TM^ tissues were treated with 3,3,5-trimethylcyclohexaneacetic acid in acetone for 48 and 72 hours, at concentrations up to 40 mg/ml. 3,3,5-Trimethylcyclohexaneacetic acid did not induce binucleated cells with micronuclei when tested up to cytotoxic levels, and therefore was concluded to be negative in the RSMN assay [86] (Fig. 21). A follow-up *in vivo* micronucleus study was also conducted in mice. The test material was administered in corn oil on two consecutive days at doses of 125, 250, or 500 mg/kg body weight via oral gavage to groups of male and female Hsd:ICR (CD-1) mice and euthanized at 48 hours. The test material did not induce a statistically significant increase in the incidence of micronucleated reticulocytes in the peripheral blood and was concluded to be negative in the *in vivo* micronucleus test [87].

### Veratraldehyde (CAS# 120-14-9)

**Figure 22:**
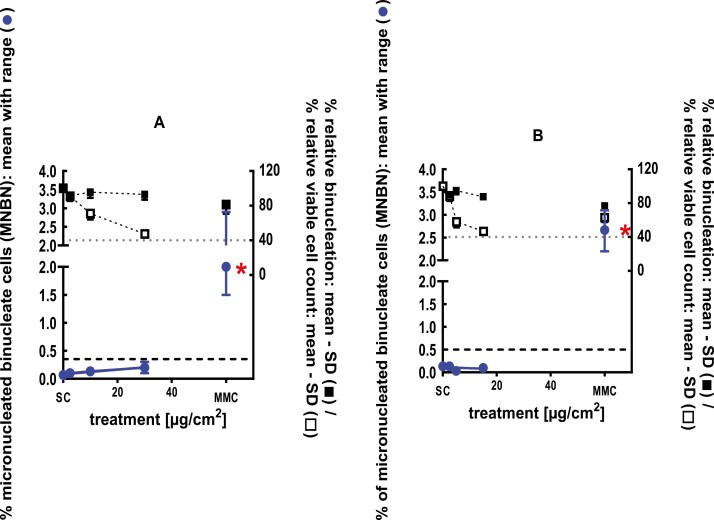
3Dskin RSMN results for Veratraldehyde (CAS#120-14-9): (A) 48-hour treatment and (B) 72-hour treatment condition. The long-dashed black line shows the lab-specific 95%-quantile of the mean of the historical control range for the MNBN data, and the thresholds for excessive cytotoxicity (40%) are shown by the black dotted line. A red asterisk indicates a statistically significant increase in % MNBN vs. the SC (Fisher’s exact test; *P* < .05). Error bars shown depict the standard error of the mean.

The clastogenic activity of veratraldehyde was evaluated in an *in vitro* micronucleus test conducted using human peripheral blood lymphocytes, at concentrations up to 1662 µg/ml in DMSO, in the presence and absence of metabolic activation. Statistically significant and dose-dependent increases in micronuclei induction were observed at a dose of 496 µg/ml in the 24-hour treatment in the absence of S9. Veratraldehyde was concluded to be positive for the induction of micronuclei in the *in vitro* mammalian cell micronucleus test [88]. A follow-up 3D skin and *in vivo* study were evaluated to verify the *in vitro* results and their biological relevance. In a GLP compliant RSMN assay, EpiDerm^TM^ tissues were treated with veratraldehyde in acetone for 48 and 72 hours, at concentrations up to 30 mg/ml. Veratraldehyde did not induce binucleated cells with micronuclei when tested up to cytotoxic levels, and therefore was concluded to be negative in the RSMN assay [89] (Fig. 22). A follow-up *in vivo* micronucleus study was also conducted in mice. The test material was administered in corn oil on two consecutive days at doses of 500, 1000, or 2000 mg/kg body weight via oral gavage to groups of male and female Hsd:ICR (CD-1) mice and euthanized at 48 hours. The test material did not induce a statistically significant increase in the incidence of micronucleated reticulocytes in the peripheral blood and was concluded to be negative in the *in vivo* micronucleus test [90].

Overall, the results for the 48-hour exposure were in agreement with the 72-hour exposure; in this set of materials both 48- and 72-hour results were negative. It is important to note that similar concentrations were evaluated in both the 48- and 72-hour exposures in this study, indicating that the 48-hour exposure does not result in the ability to test significantly higher concentrations as may be thought to be important for greater detection of genotoxicity. In fact, the 72-hour exposure has been reported as having greater sensitivity compared to the 48-hour exposure (16).

The results of the RSMN assay were in 100% agreement with the *in vivo* micronucleus assay results, where 18 chemicals positive in an *in vitro* micronucleus assay were negative in an *in vivo* micronucleus assay (Table 1).

## Discussion

The current data set comparing the standard *in vitro* and *in vivo* micronucleus assays to the RSMN assay demonstrates that the RSMN assay is an ideal model for the assessment of genotoxicity of fragrance materials. In our study, 18 fragrance materials that were positive or equivocal in the *in vitro* micronucleus test were negative in the RSMN assay which agrees 100% with the negative results in the *in vivo* micronucleus test for these materials. In addition, one material (2-ethyl-1,3,3-trimethyl-2-nobornanol) was negative in an *in vitro* micronucleus assay, negative in the RSMN, and negative in the *in vivo* micronucleus assay. Overall, the RSMN assay agreed with the *in vivo* micronucleus for 19 fragrance materials in our study. In the majority of the *in vivo* studies, the target tissue exposure was evident from the toxicity and CNS-related effects, bone marrow toxicity, or both which confirm systemic exposure.

These results support the conclusion that the RSMN assay is useful for follow-up testing of materials that are positive in standard *in vitro* tests. Positive results in *in vitro* tests for materials that are negative *in vivo*, i.e. “misleading positive result,” have been discussed extensively in the literature and are likely due to a number of factors including differences in the exogenous S9 metabolism in standard *in vitro* tests compared to *in vivo* metabolism. For example, in our dataset, the misleading positive result for 2-octen-4-one (CAS # 4643-27-0) in the *in vitro* micronucleus study in human peripheral blood lymphocytes (HPBL) could be attributed to the lack of phase-II metabolic enzyme activity which may help in detoxification of the parent and metabolite of 2-octen-4-one resulting in metabolic overload [91], or may be due to formation of a reactive oxygen species as is known to occur in alpha, beta unsaturated ketones [92], whereas 3D skin and the *in vivo* micronucleus study have phase II detoxification capability to handle oxygen species [11]. The misleading positive result in the *in vitro* micronucleus assay for 1,5-Dimethylbicyclo[3.2.1]octan-8-one-oxime may be due to the fact that oximes can form reactive sulfate conjugates that aminate DNA in the absence of appropriate metabolic transformation pathway. For example, 2-Butanone oxime (MEKO) can be directly conjugated with sulfate. Oxidation of MEKO to 2-nitrobutane has been demonstrated to occur in liver microsomal preparation [93], but likely is only a minor pathway of MEKO-biotransformation in rats *in vivo*. In rats, hydrolysis of MEKO to 2-butanone is the major pathway of biotransformation and 2-butanone is further catabolized to CO2 [94]. A final example from our dataset is for 2,2ʹ-(Dithiodimethylene)difuran and furfuryl thioacetate, where the positive outcomes in the *in vitro* micronucleus assays were only seen in shorter treatment conditions both with and without metabolic activation treatment, but it was negative in the long-term (24-hour) treatment condition in the absence of metabolic activation. For thiol compounds, the metabolism pathway seems to follow this chronology: S-oxidation, S-methylation, and fission of the disulfide bond proceeding to oxidation at the SH group of the resulting hydrolyzed compounds [95] (EFSA 2011). These can further form glutathione conjugates or undergo glucuronidation leading to its elimination. The enzymes involved in the biotransformation are primarily cytochrome P450 monooxygenase families, glycine-, glucuronide- methyl-, and glutathione transferases [95] (EFSA 2011). All these are present at sufficient level in the 3D RSMN tissue [11], hence negative results were observed in the RSMN assay and were in agreement with more biologically relevant *in vivo* studies which also has these enzymes present at sufficient levels. Taken together, our results indicate that the RSMN study generates more biologically relevant outcomes to determine genotoxic potential of fragrance materials. As such, the RSMN assay is useful for following up positive results from the standard *in vitro* cytogenetic assays.

The RSMN assay is also useful in cases where there is an equivocal or positive result that is considered non-biologically relevant in the traditional *in vitro* micronucleus assay. In the current dataset, 2,3-dihydro-1,1-dimethyl-1H-indene-ar-propanal, 5-phenylhex-3-en-2-one, and isobornyl methyl ether induced an equivocal result, whereas methyl beta-phenylglycidate and cadinene induced an increase in micronuclei in HBPL that was considered not biologically relevant (as discussed above in Results). Negative results were obtained in the RSMN assay for all of these materials, and these results agree with the negative *in vivo* micronucleus result for methyl beta-phenylglycidate and the negative read-across *in vivo* micronucleus result for isobornyl methyl ether.

Use of the RSMN assay earlier in a test battery could avoid time consuming, costly repeat testing and additional analyses that occur for those materials that induce weak or questionable effects. This is illustrated by a detailed discussion of the results of the *in vitro* micronucleus assay in HBPL for isobornyl methyl ether. Isobornyl methyl ether induced a statistically significant increase in micronuclei in the S9-activated 4-hour exposure that was dose-dependent [49]. Micronucleus induction was within the historical control range but outside the 95% historical control range, however, the Cochran-Armitage test was negative for dose–response (*P* > .05). Overall, isobornyl methyl ether was concluded to be equivocal. By contrast, isobornyl methyl ether was clearly negative in the RSMN assay. It can be argued that conducting an RSMN assay earlier in this evaluation, instead of repeating the standard *in vitro* micronucleus assay, may have resulted in an expedited, definitive assessment of a lack of genotoxicity. *In vivo*, alicyclic ethers like isobornyl methyl ether are expected to undergo either ring hydroxylation or side-chain oxidation followed by conjugation with glucuronic acid and excretion in the urine [96, 97], and therefore do not present a genotoxicity hazard.

The current study design for the RSMN assay [26] involves an initial assay using 2-day dosing regimen (48-hour treatment), and if the result is negative then a follow-up confirmatory assay using 3-day dosing regimen (72-hour treatment) is conducted [98]. In the studies reported here, consistent outcome (lack of micronuclei and similar cytotoxicity) was observed in both treatment regimens. We support the recent recommendation from the Cosmetics Europe validation of the RSMN assay by Pfuhler *et al*. to directly conduct the 72-hour treatment exclusively [16]. Considering the data pulished on the RSMN assay to date, there does not appear to be an advantage or need to routinely conduct a 2-day (48-hour) dosing regimen when there is an appropriately conducted 3-day (72-hour) RSMN assay. The RSMN study design can therefore be streamlined by conducting 3-day dosing directly and if the results are negative, the material can be concluded to be non-clastogenic. This is further supported by recent dataset comparison published by Pfuhler *et al*. which has discussed the advantages to doing a 72-hour dosing regimen [16].

## Conclusion

The RSMN assay is an important alternative to animal testing for characterization of the genotoxicity potential of fragrance materials. In the context of the results obtained here, the RSMN assay was a powerful tool to address potential misleading positive outcomes from the standard *in vitro* genotoxicity battery as it showed 100% concordance with *in vivo* outcomes. Importantly, since the primary route of exposure for fragrances is by the dermal route, the RSMN assay fits the applicability domain for testing fragrance materials.

*Conflict of interest statement*: None declared.
